# Communication of “Accoucheur”

**Published:** 1845-10-01

**Authors:** 


*To the Editor of the Buffalo Medical Journal:*

It is a trite remark, true as trite, that a trifling incident in early life often stamps the character of the man for good or ill. Soon after I "set up in business," in a country town of farmers, mostly strangers to me, I was called in haste to meet Dr. H., of a neighboring town, at the house of Mr. G., and to bring my "instruments," as Mrs. G. was very sick.—The condition of Mrs. G. was readily inferred, but my instruments! When I parted with my best friend, the very respectable physician and most excellent man, under whose iustruction I had acquired what I knew of the art, and from whom I certainly derived much practical information, with his blessing and best wishes for my future success, he told me he had a pair of Smellie's long forceps, and, as he never used but one blade, he would make a present of the other to me. With this blade, then, and it was all I had, I repaired to the house of Mr. G., where I found Dr. H., a respectable looking, elderly man of from 55 to 60 years of age, who had been in attendance on Mrs. G., in labor with her second child, for the last 36 hours. Though the labor was protracted, it was a natural one—in common parlance "all was right," without an untoward symptom, and wanted notning but a few expulsive pains.

Dr. H. proposed the instant application of the forceps, but this proposition was cautiously evaded, for reasons which the reader will be at no loss to divine. I was in no haste to exhibit my odd blade of Smellie's forceps. So, assuring the patient of her perfect safety, and of the moral certainty that in a short time the labor would terminate happily, she took some lavender in water, (ergot was unknown) and left to repose not over half an hour, when the pains returned, and in a few minutes the child was expelled. This fact was bruited far and wide, the country over, and proved to me what the passage of Lodi, or the battle of Austerlitz did to a greater man, who afterward consummated his victories and triumphs at St. Helena by quarreling with Sir Hudson Lowe about his bread and butter. Unimportant as this case really was in itself, public opinion gave it a consequence which it did not become me to gainsay—"If Fate would make me King, why Fate might crown me." It proved to me an incalculable benefit, for few cases of difficult or protracted labor occurred for years afterward within reach of me, that I was not privileged to see—and now after the lapse of more than 35 years, and that generation has passed away, "the story lives in tradition." Perhaps I may hereafter give you the history of some really interesting cases in this department, which this case introduced me to, and which I have used to introduce myself to you as an

ACCOUCHEUR.

